# Serendipitous discovery of *Wolbachia *genomes in multiple *Drosophila *species

**DOI:** 10.1186/gb-2005-6-3-r23

**Published:** 2005-02-22

**Authors:** Steven L Salzberg, Julie C Dunning Hotopp, Arthur L Delcher, Mihai Pop, Douglas R Smith, Michael B Eisen, William C Nelson

**Affiliations:** 1 The Institute for Genomic Research, 9712 Medical Center Drive, Rockville, MD 20850, USA; 2 Agencourt Bioscience Corporation, 100 Cumming Center, Beverley, MA 01915, USA; 3 Center for Integrative Genomics, University of California, Berkeley, CA 94720, USA

## Abstract

**Background:**

The Trace Archive is a repository for the raw, unanalyzed data generated by large-scale genome sequencing projects. The existence of this data offers scientists the possibility of discovering additional genomic sequences beyond those originally sequenced. In particular, if the source DNA for a sequencing project came from a species that was colonized by another organism, then the project may yield substantial amounts of genomic DNA, including near-complete genomes, from the symbiotic or parasitic organism.

**Results:**

By searching the publicly available repository of DNA sequencing trace data, we discovered three new species of the bacterial endosymbiont *Wolbachia pipientis *in three different species of fruit fly: *Drosophila ananassae*, *D. simulans*, and *D. mojavensis*. We extracted all sequences with partial matches to a previously sequenced *Wolbachia *strain and assembled those sequences using customized software. For one of the three new species, the data recovered were sufficient to produce an assembly that covers more than 95% of the genome; for a second species the data produce the equivalent of a 'light shotgun' sampling of the genome, covering an estimated 75-80% of the genome; and for the third species the data cover approximately 6-7% of the genome.

**Conclusions:**

The results of this study reveal an unexpected benefit of depositing raw data in a central genome sequence repository: new species can be discovered within this data. The differences between these three new *Wolbachia *genomes and the previously sequenced strain revealed numerous rearrangements and insertions within each lineage and hundreds of novel genes. The three new genomes, with annotation, have been deposited in GenBank.

## Abstract 2

By searching the publicly available repository of DNA sequencing trace data, we discovered three new species of the bacterial endosymbiont *Wolbachia pipientis* in three different species of fruit fly: *Drosophila ananassae*, *D. simulans*, and *D. mojavensis*.

## Background

Large-scale sequencing projects continue to generate a growing number of new genomes from an ever-wider range of species. A rarely noted and unappreciated side effect of some projects occurs when the organism being sequenced contains an intracellular endosymbiont. In some cases, the existence of the endosymbiont is unknown to both the sequencing center and the laboratory providing the source DNA. Fortunately, many genome projects deposit all their raw sequence data into a publicly available, unrestricted repository known as the Trace Archive [1]. By conducting large-scale searches of the Trace Archive, one can discover the presence of these endosymbionts and, with the aid of bioinformatics tools including genome assembly algorithms, reconstruct some or most of the endosymbiont genomes.

The amount of endosymbiont DNA present in a genome deposited in the Trace Archive depends on several factors: the number of sequences generated by the project, the size of the host genome, the size of the endosymbiont genome, and the number of copies of the endosymbiont present in each cell of the host. Because the copy number varies among cell types, the amount of endosymbiont DNA also depends on the preparation method used to extract host DNA; for example, the use of eggs or early-stage embryos will yield much greater amounts of *Wolbachia *from its hosts, because the bacterium occurs in much higher copy numbers in egg cells than in other cell types [2]. If the host genome is 200 million base-pairs (Mbp) in length, and the endosymbiont is 1 Mbp, and if there is one endosymbiont per host cell, then 0.5% of the sequences from a random sequencing project of the host will derive from the endosymbiont. The critical factor is the copy number per cell: regardless of genome size, if there is one endosymbiont genome per cell, then the endosymbiont will be sequenced to the same depth of coverage as the host, and the genome assembly will, in theory, cover both genomes to the same extent.

The search for these hidden genomes is aided greatly by the availability of a complete genome of a related species. Fortunately, the complete genome of *Wolbachia pipientis w*Mel, an endosymbiont of *D. melanogaster *[3], is available to aid the search. *Wolbachia *species are common obligate intracellular parasites that infect a wide variety of invertebrates, including not only fruit flies but also mosquitoes, arthropods and nematodes [4,5].

## Results and discussion

Using the 1,267,782 bp *w*Mel genome as a probe, we searched the Trace Archive entries of seven recently sequenced *Drosophila *species, each of which was sequenced to approximately eightfold coverage. For three of these species, we found clear evidence of *Wolbachia *infections in the host.

From the 2,772,509 traces of *Drosophila ananassae *[6], we retrieved 32,720 sequences that either matched the *w*Mel strain or were paired with sequences that matched *w*Mel (see Materials and methods). Our assembly of these sequences yielded a new genome, *Wolbachia w*Ana, containing 1,440,650 bp in 329 separate scaffolds, at approximately eightfold coverage. At this coverage depth, we estimate that 98% of the *w*Ana genome is included in the assembly. The alignment of the *w*Ana scaffolds to *w*Mel covers approximately 878 kbp (70%) of the 1.27 Mb *w*Mel genome. A mapping of all the individual *w*Ana reads to *w*Mel gives greater coverage - 1.11 Mbp (87%) of the *w*Mel genome.

From the 2,214,248 traces of *D. simulans *[7], we retrieved and assembled 3,727 sequences. The resulting genome fragments of *Wolbachia w*Sim cover 896,761 bp of *w*Sim at twofold coverage, which we estimate to cover 65-80% of *w*Sim. The comparative assembly (see Materials and methods) resulted in 388 contigs plus 241 singleton sequences, and a separate scaffolding program further grouped 273 of these contigs into 84 scaffolds. The alignment between *w*Sim and *w*Mel covers 861 kbp (65%) of the *w*Mel genome.

From the 2,445,065 traces of *D. mojavensis *[6], we retrieved 101 sequences matching *w*Mel, plus another 13 sequences that did not match *w*Mel but were paired with the matching sequences. The sample is too small for assembly, but even so it represents approximately 87 kb (6-7%) of the *Wolbachia w*Moj genome.

No *Wolbachia *sequences were found in the other Drosophila species currently available: *D. pseudoobscura*, *D. yakuba*, *D. virilis *and *D. melanogaster*.

*Wolbachia *has previously been described to infect multiple strains of *D. simulans*, and a fragment of the 16S ribosomal RNA gene has been sequenced (GenBank ID AF312372) [8]. It has also been described in *D. ananassae *[9], but has not been previously reported in *D. mojavensis *(and no sequences can be found in the *Wolbachia *database maintained at [10]).

### Genome organization

**Figure 1:**
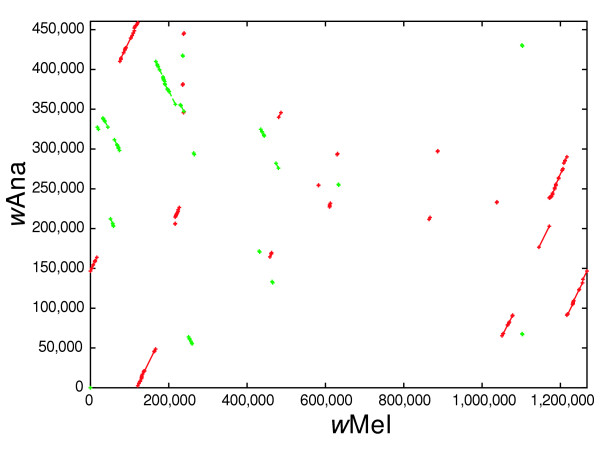
Alignment of complete *w*Mel genome (horizontal axis) to longest scaffold from the wAna genome assembly. Red points indicate sequences aligned in the forward orientation, green points indicate reverse orientation. The diagonals represent colinear regions, and breaks in the diagonals correspond to inversions and translocations between the two genomes.

Comparison of the *w*Ana and *w*Mel species indicates extensive rearrangements between the genomes. This is best illustrated with the longest scaffold in *w*Ana, which contains 455,845 bp, approximately one-third of the genome. Figure 1 shows a map of this scaffold compared to the *w*Mel genome. The scaffold spans more than a dozen rearrangements that have occurred since the divergence of these species. We also found evidence of rearrangements within our *w*Ana sequences (see Materials and methods), indicating that the *D. ananassae *strain may have been infected with two or more divergent *Wolbachia *strains. The rearrangements shown in Figure 1 are typical of the interstrain alignments; breakpoints occur even among the very sparsely sampled *w*Moj sequences. Although only 101 sequences matched *w*Mel, seven of these spanned either insertions or large-scale rearrangements in the *w*Mel genome.

### Genome comparisons

In these assemblies, approximately 464, 92 and 6 genes were discovered in the *w*Ana, *w*Sim and *w*Moj genomes, respectively (see Additional data file 1), that were not found in the previously reported *W. pipientis w*Mel genome. Of these novel genes, 343 were conserved hypothetical proteins, 81 transposases, 13 phage-related proteins and seven ankyrin domain proteins. Of the remaining 118 genes, 34 are proteins from the *w*Ana assembly of insect origin, which are likely to represent *Drosophila *contaminants as a result of chimeric inserts in the original sequencing library. Another 51 predicted genes are shorter than 300 bp and may not constitute real genes. The remaining 33 genes have similarity to known genes and include genes that have tentatively been identified to be involved in transport, DNA binding or regulation, and a variety of other functions. Many of the unique genes have anomalous GC content, suggesting horizontal gene transfer (HGT), with 12 genes displaying a GC content greater than 50% as opposed to the typical 35% GC content found in these genomes and *w*Mel (Table 1).

Consistent with the observation that novel genes in the new *Wolbachia *strains tend to be hypothetical proteins, genes present in *w*Mel that are absent in the *w*Ana assembly are also predominantly hypothetical proteins. Of the 347 *w*Mel genes not found in *w*Ana, 207 were hypothetical proteins, with the next highest category being mobile elements and extrachromosomal elements, with 37 genes. This suggests that as much as 27% of the predicted genes in *w*Mel could be highly variable.

**Figure 2:**
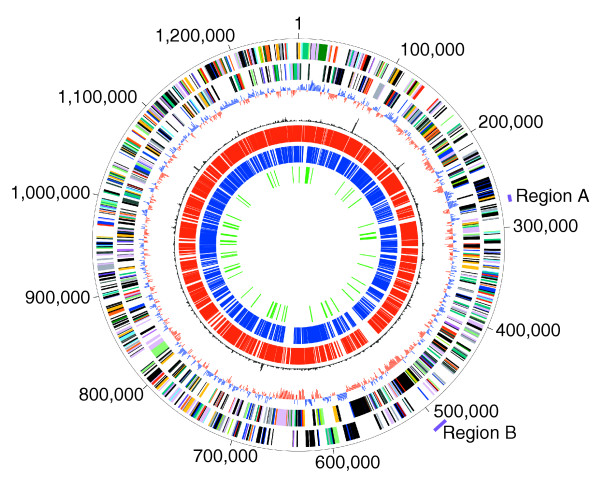
Circular map comparing the *w*Mel genome with the *w*Ana, *w*Sim and *w*Moj assemblies. Ring 1 (outermost ring): forward strand genes; ring 2: reverse strand genes; ring 3: GC-skew plot; ring 4: X^2 ^analysis of trinucleotide composition, with peaks indicating atypical regions; ring 5: *w*Mel genes present in wAna assembly; ring 6: *w*Mel genes present in the wSim assembly; ring 7: *w*Mel genes present in *w*Moj assembly. Large regions on the *w*Mel genome that were not recovered in the *w*Ana or *w*Sim assemblies are marked on the outside (regions A, B).

Two large gene clusters in *W. pipientis w*Mel were not identified in the *w*Sim and *w*Ana assemblies (Figure 2). This could suggest absence or divergence of these regions. The lack of the recovery of two of the regions (A and B) is interesting as both regions contain genes that have been suggested to affect host-endosymbiont interactions [3].

Region A includes the 3'-region of the WO-A phage and the region directly downstream. It includes the interval containing genes WD0289-WD0296, which encodes four hypothetical proteins - three ankyrin repeat domain proteins and a conserved hypothetical protein. The absence of WD0289-WD0292 is interesting because it may suggest some variation in the phage 3'-region. Although WD0289-WD00291 is unique to WO-A, a protein homologous to WD0292 has been found in the previously described *Wolbachia *phage [3,11]). Variation in the *Wolbachia *phage could facilitate the introduction of novel genes [12]. As ankyrin repeat proteins, WD0291, WD0292, and WD0294 are all of interest as they have been proposed to be involved in host-interaction functions [3]. This could provide a means by which the phage could cause different host-interaction phenotypes.

Region B includes WD0509-WD0514, which encodes a DNA mismatch repair protein MutL-2, a degenerate ribonuclease, a conserved hypothetical protein, two hypothetical proteins and an ankyrin repeat domain protein. This region is of further interest since WD0511-WD0514 is found only in *W. pipientis w*Mel and not the related sequenced Anaplasmataceae, Rickettsiaceae or α-Proteobacteria. In *W. pipientis w*Mel, this region is flanked on the 3'-end by an interrupted reverse transcriptase and an IS5 transposase, supporting the hypothesis that it was acquired horizontally. The absence of MutL-2 might not be functionally important since *w*Mel, *w*Ana, and *w*Sim all have a copy of MutL-1.

### Evolutionary comparisons

We aligned all genomes to one another to find those sequences shared by all four strains. Because *W. pipientis w*Moj comprises the smallest sample, we used the 114 sequences from that strain as a query to search the other three strains, and found 90 sequences shared among all strains. We then created four-way multi-alignments for each of these 90 sequences (see Materials and methods). Excluding the large insertions and deletions discussed above, the strains are highly similar, as summarized in Table 2.

As the table shows, the two most closely related strains are *w*Ana and *w*Sim, which are nearly identical at the DNA level. Both *w*Mel and *w*Moj are approximately equidistant from these two strains, at just over 97% identity, but are more distant from one another. Note however that because the *w*Moj sequences are single reads (that is, single-pass sequencing), the error rate in these sequences is substantially higher than in the assembled genomes of the other strains, which in turn may make it appear that *w*Moj is more divergent.

### Ankyrin repeat domain proteins

Ankyrin repeat proteins showed considerable variability among the four *Wolbachia *strains. It has been proposed that ankyrin repeat proteins may influence the host by regulating host cell cycle, regulating host cell division, and interacting with the host cytoskeleton [3]. These genes and their relationship to cell cycle, and therefore reproduction, are likely candidates for involvement in host interactions like cytoplasmic incompatibility, male killing, parthenogenesis and feminization.

**Figure 3:**
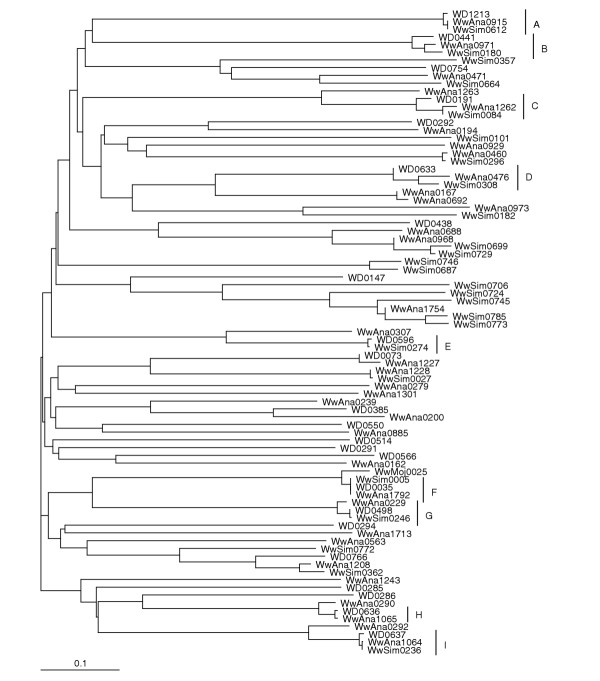
Relationship of ankyrin repeat domain proteins between *w*Mel, *w*Ana, *w*Sim and *w*Moj. All the predicted ankyrin repeat proteins with greater than 120 amino acids were aligned and clustered using ClustalW. Nine predicted ankyrin repeat domain proteins (A-I) were found to be conserved among at least *w*Mel and one other of these *Wolbachia *species with nucleotide sequence identity > 95% across the entire length of the gene.

There were four ankyrin repeat proteins absent in *w*Ana and *w*Sim in the Regions A and B above. There were also seven new ankyrin repeat proteins identified in *w*Ana, *w*Sim, and *w*Moj. In order to infer a relationship between the ankyrin repeat proteins, all the ankyrin repeat-containing proteins greater than 120 amino acids in length were aligned and clustered using ClustalW. The amino-acid sequences were too diverse to permit the construction of a reliable phylogenetic tree. But a tree was drawn that clustered similar proteins and allowed for the classification of families of conserved ankyrin repeat domain proteins within the *Wolbachia *lineage (Figure 3). From this tree, several classes of proteins can be determined that are highly conserved between two or more of these *Wolbachia *lineages with greater than 95% similarity at the nucleotide level. In addition, ankyrin repeat domain proteins unique to a particular lineage can also be identified. These differences in the complement of ankyrin repeat domain proteins may affect host-endosymbiont interactions.

### Comparison with other obligate intracellular bacteria

The variability of genome content and synteny identified here with *Wolbachia *is in contrast to that observed for other obligate intracellular bacteria. Comparative analysis of the Chlamydiaceae shows that the genomes of these organisms are highly conserved in terms of content and gene order, with relatively small differences in the genomes [13]. This is despite the fact that the chlamydial genomes sequenced thus far span four distinct species from various hosts and cause different tissue tropism and disease pathology.

Similarly, rickettsial genomes have a high degree of synteny and gene conservation with the exception of numerous unique sequences in the genome of *Rickettsia conorii *[14]. Although *R. conorii *maintains synteny with *Rickettsia prowazekii *and *Rickettsia typhi*, it has 560 unique genes relative to the other two. In contrast, the sequencing of *R. typhi *revealed only 24 novel genes.

*Wolbachia *genomes seem to have little synteny [3] and large variations in genome size and genome content. This may reflect the levels of intraspecies contact *in vivo*. *Wolbachia *are abundant in nature, are able to co-infect arthropods [15,16], and are propagated by vertical and horizontal transmission [17]. Phylogenetic analysis of the WO-B phage shows that under conditions of co-infection, *Wolbachia *from different supergroups will share the same WO-B phage [12]. These factors may promote genetic exchange between *Wolbachia *species. In addition, the *Wolbachia *lifestyle of facilitating its own transmission by host reproductive modification may then promote the successful transmission of genetically diverse strains. Other obligate intracellular bacterial genera may find the series of events involving successful co-infection, exchange of genetic information, and then propagation more challenging and therefore less likely.

### Horizontal gene transfer

The presence of endosymbionts within host cells, particularly germline cells, may offer opportunities for HGT, although in general such transfer between prokaryotes and eukaryotes is extremely rare [18]. However, a number of studies have clearly documented cases of transfer of mitochondrial DNA into the nuclear genome [19], in species as diverse as yeast [20], *Arabidopsis thaliana *[21] and other plants [22], and human [23]. The mitochondrial organelle itself is widely believed to derive from an ancestral endosymbiont [19,24]. Although we do not here provide evidence for HGT from *Wolbachia *to *Drosophila*, at least one recent study claims that a *Wolbachia *endosymbiont has transferred genes to the X chromosome of an insect, the adzuki bean beetle [25]. The analysis of the *w*Mel genome examined this question, but did not find any evidence for HGT into the *D. melanogaster *host [3].

## Conclusions

The discovery of these three new genomes demonstrates how powerful the public release of raw sequencing data can be. Although none of these projects had as its goal the sequencing of bacterial endosymbionts, we now have as a result three partial genomes - one nearly complete - of this biologically important species. The differences between these genomes and the completed *w*Mel strain demonstrate extensive genome rearrangement and divergence among these *Wolbachia *endosymbionts. And although it is a small sample, when taken together the presence of these three new genomes indicates that *Wolbachia *endosymbionts appear to be quite common in the *Drosophila *lineage. Multiple future *Drosophila *sequencing projects are planned, several of which are already underway, as are projects to sequence other invertebrates, many of which may host *Wolbachia *or other endosymbionts. Our results suggest that new screening methods, such as those described here, may yield unexpected discoveries from the data in the Trace Archive.

## Materials and methods

We downloaded from the Trace Archive at NCBI [1] the following numbers of raw sequences from each Drosophila species: 2,772,509 sequences from *D. ananassae*; 2,445,065 from *D. mojavensis*; 2,214,248 from *D. simulans*; 2,061,010 from *D. yakuba*; 3,359,782 from *D. virilis*; 2,590,703 from *D. pseudoobscura*; and 3,663,352 from *D. melanogaster*. For each project, we downloaded sequences, quality values, and ancillary data (containing clone-mate information, clone insert lengths, and sometimes trimming parameters), comprising approximately 2-3 gigabytes (GB) of compressed data per genome.

For each genome, we used the nucmer program from the MUMmer package [26-28] to search the complete genome of *W. pipientis w*Mel against the files containing the sequences. We pulled out any single sequence ('read') with at least one 30-bp exact match to *w*Mel, and with an extended match that spanned at least 65 bp. We then retrieved the 'clone mates' of each sequence: most of the reads in whole-genome sequencing projects are obtained via a double-ended shotgun method, meaning that both ends of each clone insert are sequenced. The Trace Archive contains a link to the clone mate for each read; we used this information to extract any mates that were not contained in our original screen. For example, the *D. ananassae *data yielded approximately 5,000 additional reads when we pulled in the mates from the original set.

We then assembled the *Wolbachia *reads in two different ways: with the Celera Assembler [29], treating it as a normal (*de novo*) whole-genome assembly, and with the AMOS-cmp assembler [30], which assembles a genome by mapping it onto a reference. For the reference genome we used *w*Mel. We used Celera Assembler on the relatively well-covered *w*Ana strain; although we ran it on the *w*Sim reads as well, the sequence coverage was too light to yield a good assembly. The high degree of sequence identity, at 95-100% across most regions that are shared between strains, allowed for an excellent comparative assembly of the *w*Sim strain with AMOS-cmp.

The AMOS-cmp assembly of *w*Sim contains 388 contigs plus another 241 singleton reads, covering 896,761 bp (see Table 1). The largest contig contains 16,701 bp. Note that AMOS-cmp produces contigs but not scaffolds. The contigs can easily be aligned to the reference genome to produce scaffolds, with the caveat that any rearrangements will invalidate such scaffolding information. To avoid such problems, we ordered and oriented the contigs separately with Bambus [31], a stand-alone genome scaffolding program, using only the clone-mate information from the original shotgun data. Bambus created 84 multi-contig scaffolds that joined together 273 of the 388 contigs, with the largest scaffold containing 50,851 bp and spanning (including estimated gaps) 54,207 bp.

For *w*Ana, when we compared the *de novo *and comparative assemblies, we observed that there were multiple rearrangements in the *w*Ana genome as compared to *w*Mel. Our conclusion was that a comparative assembly, which relies on the genome structure of the reference, may be less accurate than a *de novo *assembly in the presence of extensive rearrangements, so we used the latter for our analysis.

The *w*Ana assembly presented special challenges because of what appear to be a large number of rearrangements and polymorphisms within the sequences. The number of *Wolbachia *reads provided very deep coverage, which in principle should have produced a scaffold that covered nearly the entire genome. However, a large number of clone-mate links were inconsistent with one another, indicating that the reads may have been drawn from a population in which many of the individuals had genome rearrangements with respect to one another. We also found locations spanning hundreds of nucleotides where four or five individual reads had one nucleotide and the same number had a different nucleotide. These polymorphisms made it difficult to create many consistent large scaffolds. We created multiple assemblies in which we removed many of the inconsistent links, and eventually settled on the assembly presented here as the best representative of the genome possible given the diversity in the data. The *w*Ana assembly has three large scaffolds of 460 kb, 157 kb, and 121 kb respectively, with all remaining scaffolds less than 20 kb in length. We also include a list of all the individual sequences, including those not incorporated into contigs, in our Additional data files.

To annotate the resulting sets of contigs, we used Glimmer [32,33] to make initial gene calls and BLAST [34] to search those calls against a comprehensive protein database. Regions with no gene calls were searched as well in all six reading frames using Blastx.

All the predicted genes in *w*Ana, *w*Sim, and *w*Moj were searched against *w*Mel using Blastn. The results of these searches were used to determine what genes are absent in the *w*Ana, *w*Sim, and *w*Moj assemblies. DNA sequence matches at 80% identity for 80% length of the smaller of the genes were determined to be conserved and are plotted in Figure 2. Regions A and B in Figure 2 were identified in this manner. To identify the unique genes in the *w*Ana, *w*Sim, and *w*Moj assemblies, all predicted proteins were searched against the *w*Mel proteins using Blastp. Proteins in the new genomes were considered unique (or highly divergent) when the best match in *w*Mel had an E-value greater than 10^-15^.

To create the multiple alignments of the 90 sequences that were shared by all four organisms, we searched the 114 sequences in *w*Moj against the *w*Mel, *w*Ana, and *w*Sim genome assemblies, again using nucmer. We used the output of nucmer to extract from each genome the appropriate matching sequence, and we fed the results to the overlapper (hash-overlap) from the AMOS assembler [30] to generate all pairwise sequence alignments.

All ankyrin repeat domain proteins identified by automated annotation were compiled and an alignment and tree were constructed using ClustalW [35]. The ankyrin repeat domain is a degenerate repeat [36], so no attempt was made to cluster proteins where the ankyrin repeat motifs were removed.

The whole-genome shotgun assemblies, with annotation, have been deposited at DDBJ/EMBL/GenBank under the project accession AAGB00000000 (*w*Ana) and AAGC00000000 (*w*Sim). The versions described in this paper are the first versions, AAGB01000000 and AAGC01000000. The sequences and annotation for *w*Moj have consecutive accessions AY897435 through AY897548. The unassembled *w*Moj reads are also available from the Trace Archive and from the Additional data files for this paper.

## Additional data files

The following additional data is available with the online version of this paper. Additional data file 1 contains four tables: the first three list the unique genes in the *w*Ana, *w*Sim and *w*Moj genomes respectively; the fourth lists the Trace Archive identifiers for the 114 reads comprising the *w*Moj sequences from the *D. mojavensis *genome project. Additional data file 2 is a multi-fasta file containing the sequences of the 114 *w*Moj reads.

## Supplementary Material

Additional File 1 Supplementary Tables 1, 2, and 3 listing the unique genes in the *w*Ana, *w*Sim and *w*Moj genomes respectively and Supplementary Table 4 listing the Trace Archive identifiers for the 114 reads comprising the *w*Moj sequences from the *D. mojavensis *genome project Click here for file

Additional File 2 The sequences of the 114 *w*Moj reads Click here for file

## Figures and Tables

**Table 1:** Summary statistics for assemblies of the three new *Wolbachia *genomes

	wAna	wSim	wMoj	wMel
Molecule length (bp)	1,440,650	896,761	86,870	1,267,782
Scaffolds	329	84	114	1
Genes	1837	790	63	1271
Contigs	464	388	114	1
GC content (%)	35.4	35.0	34.5	35.2
Average gene length (bp)	608	916	633	855

The wSim genome was assembled using the comparative assembler, AMOS-Cmp, and scaffolded using Bambus. The wAna genome was assembled using the Celera Assembler, as described in Materials and methods. Note that the high gene count for wAna is likely due to fragmentation of individual genes across separate contigs.

**Table 2:** Percent identity between nucleotide sequences of the four sequenced strains of *Wolbachia*

	*w*Mel	*w*Ana	*w*Sim
*w*Mel		97.2	97.1
*w*Ana	97.2		99.8
*w*Sim	97.1	99.8	
*w*Moj	94.9	97.5	97.3
