# Supplementary material for: Serendipitous discovery of Wolbachia genomes in multiple Drosophila species
Source: Genome Biol. 2005 Feb 22;6(3):R23. doi: 10.1186/gb-2005-6-3-r23 (PMC1088942; doi:10.1186/gb-2005-6-3-r23)
Supplement: Additional File 1 — Supplementary Tables 1, 2, and 3 listing the unique genes in the wAna, wSim and wMoj genomes respectively and Supplementary Table 4 listing the Trace Archive identifiers for the 114 reads comprising the wMoj sequences from the D. mojavensis genome project [file gb-2005-6-3-r23-S1.doc]

SUPPLEMENTARY TABLE 1 Unique genes in *w*Ana assembly

| ORF | 5’-end | 3’end | Size | % GC | Common name |
| --- | --- | --- | --- | --- | --- |
| ORF00005 | 3606 | 3866 | 261 | 34.5 | transposase |
| ORF00006 | 3986 | 4327 | 342 | 33.6 | transposase |
| ORF00009 | 7394 | 7528 | 135 | 32.6 | prophage LambdaW4, DNA methylase |
| ORF00013 | 10458 | 10559 | 102 | 40.2 | conserved hypothetical protein |
| ORF00014 | 10768 | 10673 | 96 | 25 | conserved hypothetical protein |
| ORF00019 | 12929 | 13033 | 105 | 41 | DNA polymerase III, gamma and tau subunits |
| ORF00024 | 15609 | 15710 | 102 | 30.4 | hypothetical protein |
| ORF00029 | 19246 | 18659 | 588 | 34.9 | hypothetical transposase |
| ORF00036* | 25615 | 23888 | 1728 | 47.3 | SD27140p |
| ORF00038 | 26364 | 26498 | 135 | 35.6 | hypothetical protein |
| ORF00039 | 26485 | 26739 | 255 | 35.7 | transposase |
| ORF00043 | 28708 | 28830 | 123 | 38.2 | hypothetical protein |
| ORF00058 | 35435 | 35629 | 195 | 42.1 | hypothetical protein |
| ORF00059 | 35637 | 35768 | 132 | 38.6 | phosphoribosylglycinamide formyltransferase |
| ORF00063 | 38450 | 38325 | 126 | 30.2 | hypothetical protein |
| ORF00066 | 41135 | 41449 | 315 | 37.1 | conserved hypothetical protein |
| ORF00072 | 45950 | 46117 | 168 | 41.1 | hypothetical protein |
| ORF00074 | 47214 | 47342 | 129 | 36.4 | prophage LambdaW5, ankyrin repeat domain protein |
| ORF00075 | 47725 | 47618 | 108 | 23.1 | hypothetical protein |
| ORF00076 | 47900 | 47784 | 117 | 37.6 | prophage LambdaW5, site-specific recombinase, resolvase family |
| ORF00080 | 50483 | 50388 | 96 | 54.2 | hypothetical protein |
| ORF00081 | 50889 | 50782 | 108 | 23.1 | hypothetical protein |
| ORF00090 | 58205 | 58333 | 129 | 34.9 | ATP-dependent protease La |
| ORF00092 | 59288 | 59184 | 105 | 42.9 | hypothetical protein |
| ORF00093 | 61098 | 60256 | 843 | 37.4 | conserved hypothetical protein |
| ORF00094 | 61319 | 61167 | 153 | 24.8 | hypothetical protein |
| ORF00097 | 63516 | 63385 | 132 | 32.6 | similar to (NP 034322.1|) sex-determination protein homolog Fem1a [Mus musculus] |
| ORF00099 | 63746 | 63862 | 117 | 28.2 | hypothetical protein |
| ORF00101 | 65341 | 65234 | 108 | 49.1 | conserved hypothetical protein |
| ORF00105 | 68504 | 68403 | 102 | 30.4 | hypothetical protein |
| ORF00106 | 68893 | 68771 | 123 | 43.9 | 3-octaprenyl-4-hydroxybenzoate carboxy-lyase |
| ORF00110 | 70876 | 70971 | 96 | 35.4 | hypothetical protein |
| ORF00116 | 75600 | 75704 | 105 | 42.9 | hypothetical protein |
| ORF00128 | 82440 | 82252 | 189 | 33.3 | conserved hypothetical protein |
| ORF00131 | 85132 | 84992 | 141 | 32.6 | hypothetical protein |
| ORF00138 | 89454 | 89552 | 99 | 38.4 | hypothetical protein |
| ORF00140 | 90633 | 90743 | 111 | 29.7 | hypothetical protein |
| ORF00141 | 91521 | 91372 | 150 | 31.3 | prophage LambdaW1, transposase, IS110 family |
| ORF00143 | 92109 | 92216 | 108 | 38.9 | hypothetical protein |
| ORF00148 | 95858 | 95247 | 612 | 34.8 | hypothetical transposase, putative |
| ORF00152 | 97876 | 98100 | 225 | 34.7 | DNA-binding protein, putative |
| ORF00158 | 101045 | 101158 | 114 | 36.8 | succinate dehydrogenase, iron-sulfur protein |
| ORF00160 | 102530 | 102649 | 120 | 23.3 | hypothetical protein |
| ORF00162 | 102677 | 102871 | 195 | 34.9 | prophage P2W3, tail protein D, putative |
| ORF00163 | 103498 | 103716 | 219 | 37.4 | hypothetical protein |
| ORF00165 | 104727 | 104849 | 123 | 22.8 | hypothetical protein |
| ORF00172 | 110485 | 110634 | 150 | 32.7 | hypothetical protein |
| ORF00173 | 110631 | 110771 | 141 | 37.6 | membrane protein, putative |
| ORF00179 | 115163 | 115285 | 123 | 45.5 | conserved hypothetical protein |
| ORF00182 | 115833 | 116045 | 213 | 38.5 | ribosomal protein L14 |
| ORF00184 | 118137 | 118484 | 348 | 36.2 | DNA invertase |
| ORF00186 | 119586 | 119005 | 582 | 36.1 | transposase family protein |
| ORF00187 | 119632 | 119528 | 105 | 30.5 | hypothetical protein |
| ORF00189 | 121002 | 121133 | 132 | 28 | signal recognition particle protein |
| ORF00194 | 123446 | 123550 | 105 | 29.5 | hypothetical protein |
| ORF00198 | 130408 | 130506 | 99 | 36.4 | hypothetical protein |
| ORF00201 | 132703 | 132566 | 138 | 30.4 | hypothetical protein |
| ORF00218* | 144358 | 145101 | 744 | 46.4 | KIAA1133 protein |
| ORF00223 | 147752 | 147507 | 246 | 33.7 | transposase 1 |
| ORF00225 | 149037 | 148921 | 117 | 29.1 | metal-binding protein |
| ORF00226 | 149155 | 149054 | 102 | 28.4 | conserved hypothetical protein |
| ORF00227 | 151708 | 151821 | 114 | 48.2 | hypothetical protein |
| ORF00236 | 160339 | 160494 | 156 | 39.7 | ribosomal protein L7/L12 |
| ORF00242 | 162991 | 162815 | 177 | 25.4 | transposase, degenerate |
| ORF00243 | 162834 | 163025 | 192 | 26.6 | conserved hypothetical protein |
| ORF00249 | 165310 | 165212 | 99 | 38.4 | transposase, IS5 family, OrfB |
| ORF00250 | 165444 | 165349 | 96 | 30.2 | transposase, IS5 family, OrfB |
| ORF00255 | 168593 | 168688 | 96 | 33.3 | hypothetical protein |
| ORF00256 | 168704 | 168799 | 96 | 32.3 | hypothetical protein |
| ORF00259 | 171202 | 171321 | 120 | 30.8 | S-adenosylmethionine synthetase |
| ORF00263 | 174267 | 174395 | 129 | 34.1 | transposase, degenerate |
| ORF00264 | 174576 | 174680 | 105 | 27.6 | hypothetical protein |
| ORF00266 | 175790 | 175894 | 105 | 33.3 | conserved hypothetical protein |
| ORF00268* | 176993 | 177622 | 630 | 44.4 | Pol protein |
| ORF00277 | 184031 | 182760 | 1272 | 37.3 | transposase homolog , putative |
| ORF00278* | 184395 | 184063 | 333 | 34.5 | proteasome 26S subunit, non-ATPase, 10 |
| ORF00279 | 185037 | 184420 | 618 | 30.9 | Putative ankyrin-repeat protein, putative |
| ORF00280 | 185898 | 186167 | 270 | 34.1 | prophage LambdaW1, transposase, IS110 family |
| ORF00285 | 189828 | 189989 | 162 | 29 | membrane protein, putative |
| ORF00288 | 192126 | 192034 | 93 | 32.3 | hypothetical protein |
| ORF00296 | 196678 | 196776 | 99 | 39.4 | ISSod13, transposase |
| ORF00299 | 197760 | 197864 | 105 | 34.3 | hypothetical protein |
| ORF00300 | 197833 | 198567 | 735 | 35.4 | ISPsy5, transposase, interruption-C, putative |
| ORF00302 | 199976 | 199251 | 726 | 32.5 | hypothetical protein |
| ORF00303 | 199949 | 200110 | 162 | 35.8 | hypothetical protein |
| ORF00304 | 200172 | 200330 | 159 | 30.2 | hypothetical protein |
| ORF00309 | 203851 | 204438 | 588 | 34.9 | hypothetical transposase, putative |
| ORF00315 | 207069 | 207677 | 609 | 38.4 | hypothetical protein |
| ORF00324 | 214039 | 213944 | 96 | 34.4 | tRNA (guanine-N1)-methyltransferase |
| ORF00326 | 214789 | 214962 | 174 | 43.1 | Mg(2+) chelatase family protein BMEI1994 |
| ORF00329* | 216289 | 217632 | 1344 | 29 | pol protein |
| ORF00330* | 217895 | 218479 | 585 | 26.7 | SD03311p |
| ORF00332 | 220148 | 220288 | 141 | 38.3 | cytochrome b |
| ORF00334 | 221220 | 221495 | 276 | 36.2 | predicted amidophosphoribosyltransferases |
| ORF00337 | 223342 | 223473 | 132 | 31.1 | hypothetical protein |
| ORF00338 | 225572 | 225417 | 156 | 34.6 | glyceraldehyde 3-phosphate dehydrogenase |
| ORF00345 | 232097 | 231966 | 132 | 33.3 | ISEhe3 orfB |
| ORF00346 | 232611 | 232255 | 357 | 33.1 | transposase 1 |
| ORF00349 | 234205 | 234095 | 111 | 29.7 | hypothetical protein |
| ORF00355 | 237556 | 237915 | 360 | 29.7 | conserved hypothetical protein |
| ORF00356 | 238230 | 238817 | 588 | 34.9 | hypothetical transposase |
| ORF00362 | 241239 | 241439 | 201 | 35.3 | hypothetical protein |
| ORF00363 | 242768 | 241809 | 960 | 40.5 | hypothetical protein |
| ORF00364 | 244194 | 244391 | 198 | 31.8 | hypothetical protein |
| ORF00367 | 246240 | 246133 | 108 | 23.1 | hypothetical protein |
| ORF00373 | 251132 | 250908 | 225 | 30.7 | transposase and inactivated derivative, putative |
| ORF00374 | 251376 | 251194 | 183 | 34.4 | hypothetical protein |
| ORF00380 | 254544 | 254678 | 135 | 37.8 | phage uncharacterized protein |
| ORF00381 | 255491 | 255399 | 93 | 35.5 | hypothetical protein |
| ORF00382 | 256824 | 256498 | 327 | 33.9 | pilin gene inverting protein |
| ORF00383 | 256857 | 256967 | 111 | 36 | hypothetical protein |
| ORF00384 | 256971 | 257180 | 210 | 43.8 | conserved hypothetical protein |
| ORF00390 | 260884 | 260979 | 96 | 35.4 | membrane protein, putative |
| ORF00391 | 261059 | 261166 | 108 | 30.6 | conserved hypothetical protein |
| ORF00401 | 266761 | 266862 | 102 | 31.4 | transposase, IS5 family, truncation |
| ORF00403 | 268606 | 268812 | 207 | 28.5 | similar to transposase [Nostoc sp. PCC 7120] |
| ORF00404 | 268930 | 269313 | 384 | 35.9 | similar to transposase [Nostoc sp. PCC 7120] |
| ORF00405 | 269262 | 269420 | 159 | 42.1 | similar to transposase [Nostoc sp. PCC 7120] |
| ORF00406 | 269938 | 269807 | 132 | 25.8 | transposase, degenerate |
| ORF00408 | 271097 | 270990 | 108 | 38.9 | hypothetical protein |
| ORF00416 | 276018 | 275908 | 111 | 27 | hypothetical protein |
| ORF00427 | 282183 | 282299 | 117 | 34.2 | hypothetical protein |
| ORF00429 | 282286 | 282534 | 249 | 37.3 | transposase tnp |
| ORF00432 | 285675 | 285854 | 180 | 35 | Mg chelatase, subunit D/I family protein |
| ORF00433 | 285851 | 285955 | 105 | 42.9 | 2,3,4,5-tetrahydropyridine-2,6-dicarboxylate N-succinyltransferase |
| ORF00434 | 286302 | 286409 | 108 | 38.9 | hypothetical protein |
| ORF00438* | 289855 | 288779 | 1077 | 46.7 | gag-pol polyprotein precursor |
| ORF00439 | 289338 | 289451 | 114 | 44.7 | hypothetical protein |
| ORF00440 | 290475 | 290573 | 99 | 52.5 | hypothetical protein |
| ORF00443* | 292944 | 292549 | 396 | 42.9 | Pol |
| ORF00444* | 293269 | 292922 | 348 | 45.4 | ZNRF3 protein |
| ORF00445 | 293614 | 293739 | 126 | 33.3 | hypothetical protein |
| ORF00460* | 303219 | 302314 | 906 | 30.4 | pol protein |
| ORF00462 | 303314 | 303433 | 120 | 28.3 | hypothetical protein |
| ORF00472* | 310429 | 309128 | 1302 | 44.9 | SD27140p |
| ORF00474 | 311611 | 312021 | 411 | 45.7 | hypothetical protein |
| ORF00476 | 313127 | 313035 | 93 | 29 | hypothetical protein |
| ORF00481 | 316806 | 316663 | 144 | 39.6 | predicted amidophosphoribosyltransferases |
| ORF00482 | 316878 | 317000 | 123 | 38.2 | hypothetical protein |
| ORF00484 | 318304 | 318504 | 201 | 33.3 | transcriptional regulator, putative |
| ORF00486 | 319436 | 319552 | 117 | 23.1 | hypothetical protein |
| ORF00487 | 319665 | 319549 | 117 | 30.8 | conserved hypothetical protein |
| ORF00489 | 319870 | 319995 | 126 | 42.9 | hypothetical protein |
| ORF00492 | 321752 | 321925 | 174 | 35.6 | conserved hypothetical protein |
| ORF00499 | 326133 | 326393 | 261 | 47.1 | type IV secretion system protein VirB6 |
| ORF00514 | 334346 | 333759 | 588 | 34.9 | hypothetical transposase, putative |
| ORF00517 | 336107 | 336009 | 99 | 32.3 | hypothetical protein |
| ORF00524 | 339941 | 339825 | 117 | 29.9 | prophage LambdaW1, transposase, IS110 family |
| ORF00536 | 346570 | 346671 | 102 | 51 | conserved hypothetical protein |
| ORF00537 | 346954 | 347127 | 174 | 40.2 | hypothetical protein |
| ORF00538 | 347517 | 347407 | 111 | 33.3 | hypothetical protein |
| ORF00542 | 349542 | 349664 | 123 | 36.6 | putative transposase |
| ORF00544 | 350998 | 350441 | 558 | 55.9 | hypothetical protein |
| ORF00545 | 350463 | 350822 | 360 | 59.2 | hypothetical protein |
| ORF00546 | 352973 | 353236 | 264 | 38.3 | conserved hypothetical protein |
| ORF00547 | 353226 | 353390 | 165 | 32.1 | hypothetical protein |
| ORF00548 | 354421 | 354513 | 93 | 35.5 | hypothetical protein |
| ORF00553 | 357590 | 357697 | 108 | 30.6 | hypothetical protein |
| ORF00557* | 360670 | 361551 | 882 | 32.7 | 235 kDa rhoptry protein, putative |
| ORF00558 | 361603 | 361932 | 330 | 32.1 | transcriptional regulator |
| ORF00559 | 362397 | 362284 | 114 | 31.6 | hypothetical protein |
| ORF00560 | 361955 | 362329 | 375 | 35.5 | transcriptional regulator |
| ORF00561 | 363332 | 362721 | 612 | 39.4 | hypothetical protein |
| ORF00562 | 363399 | 364511 | 1113 | 34.4 | ankyrin 1, erythrocyte splice form 1 |
| ORF00564 | 365286 | 365032 | 255 | 42 | unnamed protein product; Similar to reverse transcriptase/maturase |
| ORF00567 | 367446 | 367315 | 132 | 36.4 | hypothetical protein |
| ORF00569 | 368150 | 368278 | 129 | 30.2 | hypothetical protein |
| ORF00572 | 369258 | 369461 | 204 | 28.4 | hypothetical protein |
| ORF00574 | 370890 | 370501 | 390 | 46.4 | hypothetical protein |
| ORF00576 | 371824 | 371663 | 162 | 29.6 | conserved hypothetical protein |
| ORF00577 | 371954 | 372079 | 126 | 22.2 | conserved hypothetical protein |
| ORF00582 | 374122 | 374271 | 150 | 33.3 | transposase, IS5 family, OrfB |
| ORF00588 | 377591 | 377761 | 171 | 26.9 | hypothetical protein |
| ORF00589* | 378845 | 378477 | 369 | 35.2 | indora |
| ORF00594 | 380788 | 380886 | 99 | 43.4 | hypothetical protein |
| ORF00601 | 387234 | 387121 | 114 | 25.4 | hypothetical protein |
| ORF00609 | 395868 | 395662 | 207 | 35.7 | hypothetical protein |
| ORF00611 | 396606 | 396767 | 162 | 27.2 | type IV secretion system protein VirB6 |
| ORF00612 | 397076 | 397174 | 99 | 34.3 | hypothetical protein |
| ORF00613 | 397161 | 397415 | 255 | 35.7 | transposase |
| ORF00614* | 399243 | 398071 | 1173 | 46.5 | Zinc knuckle domain protein |
| ORF00616 | 400163 | 400062 | 102 | 35.3 | penicillin-binding protein |
| ORF00618 | 400849 | 400941 | 93 | 38.7 | hypothetical protein |
| ORF00633 | 410553 | 410359 | 195 | 30.8 | conserved hypothetical protein |
| ORF00634 | 411589 | 411464 | 126 | 29.4 | hypothetical protein |
| ORF00636 | 412749 | 412850 | 102 | 30.4 | hypothetical protein |
| ORF00641 | 415017 | 414904 | 114 | 38.6 | hypothetical protein |
| ORF00646 | 417536 | 417396 | 141 | 35.5 | similar to GPJ of phage P2 |
| ORF00650 | 420011 | 419835 | 177 | 33.3 | glyceraldehyde 3-phosphate dehydrogenase |
| ORF00652 | 420903 | 421184 | 282 | 31.9 | hypothetical protein |
| ORF00653 | 421441 | 421232 | 210 | 32.9 | hypothetical protein |
| ORF00657* | 424523 | 423678 | 846 | 54.3 | gag protein |
| ORF00663 | 427710 | 427850 | 141 | 27.7 | DNA-directed RNA polymerase, alpha subunit |
| ORF00666 | 431321 | 431091 | 231 | 42 | hypothetical protein |
| ORF00669 | 433632 | 433739 | 108 | 46.3 | thioredoxin-disulfide reductase |
| ORF00670 | 433823 | 434248 | 426 | 35.4 | hypothetical protein |
| ORF00672* | 434935 | 435714 | 780 | 46.7 | SD27140p |
| ORF00673 | 436269 | 436156 | 114 | 38.6 | seryl-tRNA synthetase |
| ORF00675 | 437297 | 437395 | 99 | 33.3 | hypothetical protein |
| ORF00680 | 441461 | 441562 | 102 | 37.3 | hypothetical protein |
| ORF00682* | 444133 | 442631 | 1503 | 31.9 | pol protein |
| ORF00683 | 444114 | 444236 | 123 | 29.3 | hypothetical protein |
| ORF00690 | 449865 | 449782 | 84 | 9.5 | hypothetical protein |
| ORF00692 | 451019 | 451141 | 123 | 26 | hypothetical protein |
| ORF00693 | 451224 | 451673 | 450 | 30.9 | hypothetical protein |
| ORF00698 | 454639 | 454532 | 108 | 39.8 | hypothetical protein |
| ORF00702 | 458057 | 457926 | 132 | 32.6 | type IV secretion system protein VirB6 |
| ORF00705 | 459307 | 459405 | 99 | 34.3 | hypothetical protein |
| ORF00711 | 463714 | 463592 | 123 | 24.4 | hypothetical protein |
| ORF00714* | 464878 | 465507 | 630 | 44.1 | pol protein |
| ORF00715 | 465656 | 465784 | 129 | 45.7 | hypothetical protein |
| ORF00716 | 465850 | 466584 | 735 | 35.2 | hypothetical transposase, putative |
| ORF00723 | 470601 | 470443 | 159 | 37.1 | hypothetical protein |
| ORF00724 | 470711 | 470818 | 108 | 34.3 | conserved hypothetical protein |
| ORF00729 | 474177 | 473839 | 339 | 31.9 | conserved hypothetical protein |
| ORF00734 | 476833 | 477177 | 345 | 27.5 | Endonuclease |
| ORF00737 | 479326 | 478829 | 498 | 30.7 | hypothetical protein |
| ORF00739 | 479722 | 479958 | 237 | 33.8 | putative transposase |
| ORF00741 | 482544 | 482218 | 327 | 36.4 | conserved hypothetical protein |
| ORF00745* | 485646 | 486710 | 1065 | 48.2 | Pol protein |
| ORF00746 | 486850 | 486981 | 132 | 32.6 | similar to (NP 034322.1|) sex-determination protein homolog Fem1a [Mus musculus] |
| ORF00747 | 487254 | 487430 | 177 | 39 | hypothetical protein |
| ORF00754 | 493054 | 492878 | 177 | 36.2 | hypothetical protein |
| ORF00755 | 492834 | 492929 | 96 | 36.5 | competence lipoprotein ComL, putative |
| ORF00762 | 498315 | 498470 | 156 | 35.3 | hypothetical protein |
| ORF00763 | 499216 | 498707 | 510 | 45.9 | hypothetical protein |
| ORF00764 | 498735 | 498842 | 108 | 46.3 | hypothetical protein |
| ORF00765 | 501917 | 501783 | 135 | 31.9 | hypothetical protein |
| ORF00780 | 511367 | 511696 | 330 | 39.1 | SWIM zinc finger family |
| ORF00787 | 518927 | 519091 | 165 | 27.9 | hypothetical protein |
| ORF00797 | 526187 | 526360 | 174 | 33.9 | hypothetical protein |
| ORF00800 | 528736 | 528281 | 456 | 34.6 | heat shock protein, class I |
| ORF00804 | 532938 | 531085 | 1854 | 34.5 | Ankyrin 3 (ANK-3) (Ankyrin G) |
| ORF00829 | 554050 | 554205 | 156 | 42.9 | hypothetical protein |
| ORF00835 | 564864 | 564718 | 147 | 38.8 | hypothetical protein |
| ORF00836 | 564941 | 564801 | 141 | 36.9 | hypothetical protein |
| ORF00839 | 566209 | 566108 | 102 | 25.5 | hypothetical protein |
| ORF00847 | 572095 | 572229 | 135 | 31.1 | transposase |
| ORF00848 | 572226 | 572360 | 135 | 35.6 | conserved hypothetical protein |
| ORF00849 | 572420 | 572566 | 147 | 33.3 | hypothetical protein |
| ORF00850 | 572744 | 572848 | 105 | 42.9 | hypothetical protein |
| ORF00851 | 573385 | 572990 | 396 | 42.9 | conserved hypothetical protein |
| ORF00852* | 574695 | 573385 | 1311 | 43.9 | Zinc finger, C3HC4 type (RING finger) domain protein |
| ORF00854 | 576055 | 575495 | 561 | 36.7 | transposase, truncated |
| ORF00855 | 576434 | 576108 | 327 | 33 | similar to transposase [Nostoc sp. PCC 7120] |
| ORF00862 | 582813 | 583163 | 351 | 37.6 | conserved hypothetical protein |
| ORF00867 | 587899 | 587991 | 93 | 40.9 | hypothetical protein |
| ORF00879 | 597216 | 597434 | 219 | 27.9 | transposase, degenerate |
| ORF00880* | 597839 | 597435 | 405 | 29.1 | Prgag-pol, putative |
| ORF00881* | 599227 | 597893 | 1335 | 38.1 | gag protein |
| ORF00882 | 599849 | 599727 | 123 | 18.7 | hypothetical protein |
| ORF00886 | 604919 | 605653 | 735 | 31.6 | amino acid ABC transporter, periplasmic amino acid-binding protein, putative |
| ORF00887 | 605653 | 606300 | 648 | 31.8 | amino acid ABC transporter, permease protein SP1502 |
| ORF00893 | 613306 | 612179 | 1128 | 33.2 | conserved hypothetical protein |
| ORF00896 | 614741 | 614842 | 102 | 34.3 | hypothetical protein |
| ORF00910 | 628754 | 628647 | 108 | 25.9 | sensor histidine kinase/response regulator |
| ORF00931 | 649820 | 649608 | 213 | 36.6 | conserved hypothetical protein |
| ORF00932 | 650116 | 649817 | 300 | 33 | hypothetical protein |
| ORF00936 | 655019 | 654849 | 171 | 43.9 | hypothetical protein |
| ORF00964 | 683910 | 684035 | 126 | 34.1 | conserved hypothetical protein |
| ORF00974 | 696579 | 696460 | 120 | 39.2 | conserved hypothetical protein |
| ORF00975 | 696775 | 696650 | 126 | 28.6 | conserved hypothetical protein |
| ORF00981 | 701594 | 701460 | 135 | 34.1 | hypothetical protein |
| ORF00982 | 701767 | 701985 | 219 | 31.1 | type IV secretion system protein VirB4, putative |
| ORF00984 | 702975 | 702574 | 402 | 38.3 | putative transposase |
| ORF00992 | 707417 | 707530 | 114 | 23.7 | hypothetical protein |
| ORF00993 | 708776 | 708630 | 147 | 22.4 | hypothetical protein |
| ORF00994 | 709505 | 708954 | 552 | 39.3 | hypothetical protein |
| ORF00996 | 710919 | 711026 | 108 | 31.5 | hypothetical protein |
| ORF00997 | 711182 | 712657 | 1476 | 33.1 | hypothetical protein |
| ORF00998 | 712690 | 713934 | 1245 | 35 | hypothetical protein |
| ORF01003 | 717201 | 717064 | 138 | 42.8 | conserved hypothetical protein |
| ORF01005 | 719507 | 718398 | 1110 | 32.8 | hypothetical protein |
| ORF01006 | 718895 | 719005 | 111 | 37.8 | hypothetical protein |
| ORF01019 | 732298 | 732399 | 102 | 31.4 | transposase, IS5 family, truncation |
| ORF01026 | 736862 | 736954 | 93 | 32.3 | hypothetical protein |
| ORF01030 | 741192 | 741043 | 150 | 31.3 | hypothetical protein |
| ORF01042 | 752124 | 751999 | 126 | 32.5 | hypothetical protein |
| ORF01043 | 752164 | 752045 | 120 | 36.7 | hypothetical protein |
| ORF01046 | 753247 | 752951 | 297 | 39.4 | Major tail sheath protein FI |
| ORF01047 | 754097 | 753384 | 714 | 38.4 | Major tail sheath protein FI |
| ORF01049* | 755825 | 758947 | 3123 | 45.1 | Zinc knuckle domain protein |
| ORF01051 | 759526 | 760248 | 723 | 34.3 | hypothetical protein |
| ORF01061 | 767998 | 767891 | 108 | 38.9 | hypothetical protein |
| ORF01073 | 778052 | 777768 | 285 | 37.5 | hypothetical protein |
| ORF01075 | 778528 | 778040 | 489 | 32.1 | ISSod10, transposase OrfA, putative |
| ORF01084 | 786371 | 786048 | 324 | 32.4 | hypothetical protein |
| ORF01087 | 787030 | 786875 | 156 | 32.7 | similar to (NP 034322.1|) sex-determination protein homolog Fem1a [Mus musculus] |
| ORF01090 | 789988 | 789809 | 180 | 35 | exopolysaccharide synthesis protein ExoD-related protein |
| ORF01091 | 789730 | 789861 | 132 | 37.9 | hypothetical protein |
| ORF01092 | 790008 | 790124 | 117 | 31.6 | transposase, IS5 family, OrfB |
| ORF01094 | 791481 | 791576 | 96 | 35.4 | hypothetical protein |
| ORF01099 | 796534 | 796626 | 93 | 36.6 | conserved hypothetical protein |
| ORF01111* | 809849 | 806487 | 3363 | 47.8 | SD27140p |
| ORF01119 | 816963 | 817079 | 117 | 32.5 | hypothetical protein |
| ORF01122 | 817484 | 817359 | 126 | 32.5 | hypothetical protein |
| ORF01133 | 829668 | 829928 | 261 | 29.9 | ribosomal protein L33-related protein |
| ORF01138 | 835148 | 835056 | 93 | 26.9 | conserved hypothetical protein |
| ORF01145 | 841494 | 842954 | 1461 | 34.7 | bis(5`-nucleosyl)-tetraphosphatase, symmetrical/Trk system potassium uptake protein TrkG, fusion, putative |
| ORF01149 | 845904 | 846005 | 102 | 35.3 | conserved hypothetical protein |
| ORF01150 | 846573 | 846328 | 246 | 33.7 | IS3 transposase |
| ORF01151 | 847040 | 846747 | 294 | 34.7 | transposase tnp |
| ORF01152* | 849024 | 847177 | 1848 | 43.8 | gag-pol polyprotein |
| ORF01153* | 849806 | 848970 | 837 | 47.1 | gag-pol polyprotein, putative |
| ORF01155 | 850420 | 850307 | 114 | 25.4 | hypothetical protein |
| ORF01162 | 856581 | 855673 | 909 | 34.3 | hypothetical protein |
| ORF01163 | 856295 | 856420 | 126 | 42.9 | hypothetical protein |
| ORF01176 | 864311 | 864607 | 297 | 39.7 | hypothetical protein |
| ORF01177 | 865154 | 865330 | 177 | 37.3 | hypothetical protein |
| ORF01187 | 874776 | 875573 | 798 | 42.9 | hypothetical protein |
| ORF01188 | 876903 | 876754 | 150 | 50.7 | hypothetical protein |
| ORF01189 | 877530 | 877126 | 405 | 60.5 | hypothetical protein |
| ORF01194 | 882834 | 882526 | 309 | 32 | hypothetical protein |
| ORF01195 | 884091 | 883993 | 99 | 34.3 | hypothetical protein |
| ORF01198 | 887090 | 887839 | 750 | 35.1 | hypothetical protein |
| ORF01209 | 897609 | 897355 | 255 | 39.6 | hypothetical protein |
| ORF01215 | 902125 | 901880 | 246 | 33.7 | transposase 1 |
| ORF01216 | 902571 | 902299 | 273 | 35.2 | hypothetical protein |
| ORF01217 | 902656 | 902558 | 99 | 34.3 | hypothetical protein |
| ORF01230 | 912500 | 912697 | 198 | 39.4 | uncharacterized phage protein, putative |
| ORF01236 | 917454 | 916966 | 489 | 32.1 | ISSod10, transposase OrfA, putative |
| ORF01239 | 918777 | 918872 | 96 | 38.5 | similar to GPJ of phage P2 |
| ORF01242 | 921221 | 921502 | 282 | 36.9 | hypothetical protein |
| ORF01250 | 928034 | 928132 | 99 | 39.4 | hypothetical protein |
| ORF01253 | 930832 | 930936 | 105 | 35.2 | conserved hypothetical protein |
| ORF01255 | 932544 | 932386 | 159 | 36.5 | conserved hypothetical protein |
| ORF01258 | 934908 | 935306 | 399 | 31.1 | hypothetical protein |
| ORF01268 | 948605 | 948736 | 132 | 40.9 | hypothetical protein |
| ORF01269 | 948813 | 948911 | 99 | 39.4 | hypothetical protein |
| ORF01276 | 955699 | 955842 | 144 | 34 | hypothetical protein |
| ORF01277 | 956017 | 956148 | 132 | 37.1 | hypothetical protein |
| ORF01280 | 958331 | 958465 | 135 | 30.4 | transposase |
| ORF01281 | 958462 | 958596 | 135 | 34.8 | conserved hypothetical protein |
| ORF01282 | 958656 | 958802 | 147 | 33.3 | hypothetical protein |
| ORF01283 | 958819 | 958971 | 153 | 34 | transposase BMEII0228 |
| ORF01287 | 962625 | 962762 | 138 | 31.9 | transposase, IS4 family |
| ORF01293 | 969540 | 968998 | 543 | 35.5 | ISPsy5, transposase, interruption-C, putative |
| ORF01298 | 976892 | 976431 | 462 | 43.7 | hypothetical protein |
| ORF01299* | 978414 | 979622 | 1209 | 46 | envelope protein |
| ORF01300 | 980083 | 980253 | 171 | 26.9 | conserved hypothetical protein |
| ORF01301 | 980255 | 982507 | 2253 | 36.5 | kinesin light chain |
| ORF01307 | 987874 | 987972 | 99 | 34.3 | hypothetical protein |
| ORF01308 | 987959 | 988165 | 207 | 34.3 | transposase tnp |
| ORF01309 | 988173 | 988340 | 168 | 30.4 | hypothetical protein |
| ORF01314 | 992151 | 992354 | 204 | 38.7 | transposase |
| ORF01315 | 992370 | 992516 | 147 | 29.9 | transposase |
| ORF01317 | 993910 | 993698 | 213 | 36.2 | transposase, IS5 family, OrfB |
| ORF01320 | 995153 | 995266 | 114 | 32.5 | hypothetical protein |
| ORF01321 | 995492 | 995599 | 108 | 38.9 | hypothetical protein |
| ORF01328 | 1001913 | 1002497 | 585 | 34.4 | predicted amidophosphoribosyltransferases |
| ORF01332* | 1007658 | 1006066 | 1593 | 46.2 | Zinc knuckle domain protein |
| ORF01335 | 1009140 | 1009003 | 138 | 32.6 | DnaJ domain protein |
| ORF01336 | 1008895 | 1009050 | 156 | 32.7 | hypothetical protein |
| ORF01341 | 1012044 | 1012229 | 186 | 31.2 | hypothetical protein |
| ORF01342 | 1012253 | 1012975 | 723 | 33.5 | hypothetical protein |
| ORF01343 | 1013145 | 1013249 | 105 | 32.4 | hypothetical protein |
| ORF01348 | 1017289 | 1017396 | 108 | 25.9 | hypothetical protein |
| ORF01349 | 1017978 | 1017871 | 108 | 29.6 | hypothetical protein |
| ORF01355 | 1025132 | 1025233 | 102 | 35.3 | conserved hypothetical protein |
| ORF01356 | 1025801 | 1025556 | 246 | 33.7 | transposase 1 |
| ORF01357 | 1026268 | 1025975 | 294 | 34.7 | transposase tnp |
| ORF01362 | 1029659 | 1029754 | 96 | 43.8 | hypothetical protein |
| ORF01373 | 1039475 | 1041850 | 2376 | 31.1 | hypothetical protein |
| ORF01377 | 1044686 | 1044952 | 267 | 42.7 | hypothetical protein |
| ORF01386 | 1051854 | 1051991 | 138 | 40.6 | phage uncharacterized protein |
| ORF01387 | 1052253 | 1052345 | 93 | 40.9 | hypothetical protein |
| ORF01389 | 1053115 | 1052711 | 405 | 37.3 | hypothetical protein |
| ORF01391 | 1053314 | 1053454 | 141 | 29.8 | major facilitator family transporter |
| ORF01405 | 1062008 | 1062535 | 528 | 34.8 | transposase homolog , putative |
| ORF01413 | 1070228 | 1070407 | 180 | 36.1 | hypothetical protein |
| ORF01426 | 1082467 | 1082255 | 213 | 37.1 | conserved hypothetical protein |
| ORF01428 | 1083302 | 1083568 | 267 | 38.2 | transposase, degenerate |
| ORF01446 | 1103736 | 1102702 | 1035 | 34.3 | hypothetical protein |
| ORF01450 | 1106453 | 1106824 | 372 | 37.1 | dihydroneopterin aldolase |
| ORF01457 | 1110359 | 1110541 | 183 | 34.4 | hypothetical protein |
| ORF01458 | 1110603 | 1110827 | 225 | 30.7 | conserved hypothetical protein |
| ORF01459 | 1110841 | 1111347 | 507 | 35.9 | transposase family protein |
| ORF01464 | 1115230 | 1115376 | 147 | 30.6 | co-chaperone Hsc20 |
| ORF01472 | 1124249 | 1123008 | 1242 | 38.3 | conserved hypothetical protein |
| ORF01474 | 1124633 | 1124914 | 282 | 32.6 | ISSod12, transposase, putative |
| ORF01479 | 1128464 | 1129300 | 837 | 35.1 | transposase homolog , putative |
| ORF01495 | 1148087 | 1148227 | 141 | 39 | hypothetical protein |
| ORF01508 | 1159955 | 1160077 | 123 | 35.8 | conserved hypothetical protein |
| ORF01509 | 1160875 | 1160663 | 213 | 26.8 | transposase, degenerate |
| ORF01513 | 1163302 | 1163090 | 213 | 32.9 | tRNA (guanine-N(7)-)-methyltransferase (tRNA(m7G46)-methyltransferase) |
| ORF01540 | 1186876 | 1186736 | 141 | 36.9 | hypothetical protein |
| ORF01542 | 1189072 | 1188896 | 177 | 36.2 | conserved hypothetical protein |
| ORF01545 | 1190966 | 1191064 | 99 | 34.3 | hypothetical protein |
| ORF01546 | 1191051 | 1191323 | 273 | 35.5 | hypothetical protein |
| ORF01547 | 1191713 | 1192669 | 957 | 34.9 | transposase family protein |
| ORF01557 | 1198006 | 1197527 | 480 | 32.7 | PQQ enzyme repeat family protein |
| ORF01561 | 1201475 | 1199985 | 1491 | 33.9 | Archaeal ATPase family |
| ORF01564 | 1204769 | 1204074 | 696 | 37.9 | lysozyme |
| ORF01569 | 1208885 | 1209058 | 174 | 32.2 | hypothetical protein |
| ORF01573 | 1213392 | 1213249 | 144 | 36.1 | hypothetical protein |
| ORF01575 | 1214518 | 1214342 | 177 | 38.4 | hypothetical protein |
| ORF01584 | 1222237 | 1222130 | 108 | 32.4 | hypothetical protein |
| ORF01589 | 1223970 | 1224122 | 153 | 35.9 | conserved hypothetical protein |
| ORF01591 | 1225460 | 1225567 | 108 | 29.6 | prophage LambdaW1, transposase, IS110 family |
| ORF01595 | 1231859 | 1232119 | 261 | 34.1 | transposase, putative |
| ORF01596 | 1232193 | 1232453 | 261 | 27.6 | putative transposase |
| ORF01601 | 1236550 | 1236251 | 300 | 32 | sodium/proton antiporter |
| ORF01602 | 1237070 | 1236543 | 528 | 34.7 | Na+/H+ antiporter, MnhA component, putative |
| ORF01604 | 1237521 | 1237366 | 156 | 27.6 | Multiple resistance and pH regulation protein F (MrpF / PhaF) superfamily |
| ORF01629 | 1266153 | 1266040 | 114 | 42.1 | signal recognition particle protein |
| ORF01631 | 1267602 | 1267964 | 363 | 45.5 | hypothetical protein |
| ORF01632 | 1267968 | 1268231 | 264 | 30.3 | hypothetical protein |
| ORF01638 | 1271957 | 1272433 | 477 | 33.1 | RDD family, putative |
| ORF01642 | 1275699 | 1275980 | 282 | 49.6 | hypothetical protein |
| ORF01643 | 1275992 | 1276102 | 111 | 34.2 | fragment of transposase's ORF2 |
| ORF01646 | 1278708 | 1278944 | 237 | 34.2 | transposase |
| ORF01647 | 1279062 | 1279388 | 327 | 33.9 | prophage LambdaW1, transposase, IS110 family |
| ORF01650 | 1281610 | 1281434 | 177 | 33.9 | hypothetical protein |
| ORF01651 | 1281695 | 1281597 | 99 | 31.3 | hypothetical protein |
| ORF01655 | 1285327 | 1284041 | 1287 | 34.3 | hypothetical protein |
| ORF01658 | 1288714 | 1288568 | 147 | 33.3 | hypothetical protein |
| ORF01659 | 1288908 | 1288774 | 135 | 34.8 | hypothetical protein |
| ORF01660 | 1289039 | 1288905 | 135 | 31.1 | transposase |
| ORF01663 | 1291779 | 1291579 | 201 | 36.8 | hypothetical protein |
| ORF01669 | 1296290 | 1295931 | 360 | 37.8 | conserved hypothetical protein |
| ORF01672 | 1298856 | 1298665 | 192 | 30.7 | hypothetical protein |
| ORF01687 | 1313816 | 1313995 | 180 | 40.6 | IS630-Spn1, transposase Orf1, putative |
| ORF01688 | 1314109 | 1314276 | 168 | 33.3 | hypothetical protein |
| ORF01689 | 1315317 | 1315054 | 264 | 35.2 | transposase tnp |
| ORF01690 | 1315402 | 1315304 | 99 | 34.3 | hypothetical protein |
| ORF01691 | 1315551 | 1315366 | 186 | 37.6 | transposase, IS256 family |
| ORF01692 | 1316180 | 1316043 | 138 | 31.9 | transposase, IS4 family |
| ORF01699 | 1322499 | 1322233 | 267 | 42.7 | hypothetical protein |
| ORF01702 | 1324826 | 1325203 | 378 | 30.7 | conserved hypothetical protein |
| ORF01704 | 1327507 | 1327151 | 357 | 31.1 | hypothetical protein |
| ORF01706 | 1328878 | 1328711 | 168 | 31 | hypothetical protein |
| ORF01707* | 1330176 | 1329010 | 1167 | 28.7 | putative retrovirus-like env glycoprotein |
| ORF01708* | 1332026 | 1330194 | 1833 | 31.8 | reverse transcriptase polyprotein |
| ORF01720 | 1338226 | 1338047 | 180 | 35 | Mg chelatase, subunit D/I family protein |
| ORF01721 | 1338458 | 1338147 | 312 | 30.1 | conserved hypothetical protein |
| ORF01722* | 1338550 | 1339218 | 669 | 43.3 | indora |
| ORF01723 | 1339363 | 1339461 | 99 | 38.4 | hypothetical protein |
| ORF01727* | 1345903 | 1346661 | 759 | 45.7 | nucleic-acid binding protein, putative |
| ORF01730* | 1349060 | 1350451 | 1392 | 46.4 | Pol protein |
| ORF01731* | 1350417 | 1351145 | 729 | 45.5 | Pol protein |
| ORF01735 | 1354228 | 1354109 | 120 | 48.3 | hypothetical protein |
| ORF01746 | 1361591 | 1361689 | 99 | 32.3 | hypothetical protein |
| ORF01748 | 1362950 | 1363495 | 546 | 35.3 | transposase homolog , putative |
| ORF01755 | 1373743 | 1374120 | 378 | 31.5 | hypothetical protein |
| ORF01766 | 1386827 | 1386949 | 123 | 24.4 | hypothetical protein |
| ORF01768 | 1387837 | 1387655 | 183 | 27.9 | hypothetical protein |
| ORF01771 | 1389867 | 1389962 | 96 | 29.2 | pmbA protein |
| ORF01772 | 1390103 | 1390011 | 93 | 11.8 | hypothetical protein |
| ORF01773 | 1390336 | 1391340 | 1005 | 42 | transposase |
| ORF01774 | 1391423 | 1391515 | 93 | 11.8 | hypothetical protein |
| ORF01779 | 1397099 | 1398109 | 1011 | 34.5 | DnaJ domain protein |
| ORF01790 | 1410582 | 1410674 | 93 | 32.3 | hypothetical protein |
| ORF01794 | 1415337 | 1417316 | 1980 | 36.2 | hypothetical protein |
| ORF01795 | 1417772 | 1417647 | 126 | 46 | hypothetical protein |
| ORF01796 | 1418563 | 1418297 | 267 | 42.7 | hypothetical protein |
| ORF01797 | 1418214 | 1418789 | 576 | 39.2 | hypothetical protein |
| ORF01804 | 1425032 | 1425151 | 120 | 35.8 | ISSod13, transposase |
| ORF01808 | 1428075 | 1428218 | 144 | 34 | hypothetical protein |
| ORF01810 | 1430805 | 1430548 | 258 | 35.7 | transposase tnp |
| ORF01811 | 1430890 | 1430792 | 99 | 34.3 | hypothetical protein |
| ORF01812 | 1430921 | 1431034 | 114 | 28.1 | conserved hypothetical protein |
| ORF01822 | 1443307 | 1441841 | 1467 | 33.7 | putative POT family peptide transport protein |
| ORF01835 | 1459542 | 1459649 | 108 | 33.3 | hypothetical protein |
| ORF01837 | 1460355 | 1460504 | 150 | 36 | protein-export membrane protein SecF |

*Predicted genes in the *w*Ana assembly that appear to be of Drosophila origin.

SUPPLEMENTARY TABLE 2 Unique genes in the *w*Sim assembly

| ORF | 5’-end | 3’end | Size | % GC | Common name |
| --- | --- | --- | --- | --- | --- |
| ORF00009 | 11269 | 10547 | 723 | 32.1 | hypothetical protein |
| ORF00041 | 68382 | 68909 | 528 | 38.1 | hypothetical protein |
| ORF00107 | 164607 | 165308 | 702 | 41.5 | serine-rich protein, putative |
| ORF00109 | 167366 | 167974 | 609 | 43 | hypothetical protein |
| ORF00120 | 184162 | 183785 | 378 | 45.2 | hypothetical protein |
| ORF00150 | 227890 | 228402 | 513 | 39.4 | hypothetical protein |
| ORF00158 | 241341 | 240331 | 1011 | 37.6 | peptide chain release factor 2 |
| ORF00160 | 244457 | 243207 | 1251 | 35.1 | potassium uptake protein TrkH |
| ORF00165 | 250247 | 249393 | 855 | 30.3 | putative POT family peptide transport protein |
| ORF00187 | 284053 | 283646 | 408 | 39.2 | hypothetical protein |
| ORF00197 | 298710 | 298303 | 408 | 39.7 | NADH dehydrogenase subunit 5, putative |
| ORF00198 | 298261 | 298872 | 612 | 37.9 | conserved hypothetical protein |
| ORF00201 | 303462 | 302992 | 471 | 37.4 | hypothetical protein |
| ORF00208 | 311264 | 310770 | 495 | 33.5 | hypothetical protein |
| ORF00217 | 319969 | 319574 | 396 | 38.6 | hypothetical protein |
| ORF00226 | 333341 | 332826 | 516 | 39.5 | hypothetical protein |
| ORF00238 | 348312 | 348821 | 510 | 36.1 | hypothetical protein |
| ORF00247 | 364017 | 363496 | 522 | 37.9 | hypothetical protein |
| ORF00253 | 370275 | 369745 | 531 | 31.8 | hypothetical protein |
| ORF00263 | 381077 | 380118 | 960 | 37.5 | Phage tail sheath protein |
| ORF00276 | 395781 | 395200 | 582 | 35.4 | hypothetical protein |
| ORF00308 | 438309 | 439187 | 879 | 35.8 | hypothetical protein |
| ORF00313 | 445113 | 444586 | 528 | 29.7 | conserved hypothetical protein |
| ORF00320 | 454583 | 454038 | 546 | 36.1 | hypothetical protein |
| ORF00321 | 454093 | 454608 | 516 | 35.5 | transposase |
| ORF00333 | 469112 | 469792 | 681 | 34.8 | transposase homolog , putative |
| ORF00359 | 504154 | 503669 | 486 | 36.2 | hypothetical protein |
| ORF00381 | 536223 | 535705 | 519 | 41 | hypothetical protein |
| ORF00398 | 557038 | 557559 | 522 | 43.5 | hypothetical protein |
| ORF00415 | 591401 | 590874 | 528 | 41.1 | hypothetical protein |
| ORF00429 | 608779 | 609339 | 561 | 36.5 | similar to transposase [Nostoc sp. PCC 7120] |
| ORF00450 | 646052 | 645501 | 552 | 37.9 | hypothetical protein |
| ORF00453 | 649585 | 648974 | 612 | 35 | conserved hypothetical protein |
| ORF00459 | 657068 | 657655 | 588 | 36.6 | hypothetical protein |
| ORF00494 | 708147 | 708605 | 459 | 32.9 | hypothetical protein |
| ORF00510 | 726496 | 726966 | 471 | 37.6 | hypothetical protein |
| ORF00546 | 771938 | 772426 | 489 | 35.6 | hypothetical protein |
| ORF00575 | 812558 | 812115 | 444 | 38.3 | hypothetical protein |
| ORF00577 | 813606 | 813016 | 591 | 37.1 | hypothetical protein |
| ORF00590 | 831124 | 831720 | 597 | 33.3 | DnaJ domain protein |
| ORF00627 | 874590 | 875147 | 558 | 39.4 | conserved hypothetical protein |
| ORF00635 | 888667 | 888140 | 528 | 34.7 | conserved hypothetical protein |
| ORF00656 | 917330 | 916758 | 573 | 36.1 | hypothetical protein |
| ORF00657 | 917948 | 918814 | 867 | 31.9 | hypothetical protein |
| ORF00671 | 932530 | 933393 | 864 | 35.3 | hypothetical protein |
| ORF00672 | 934988 | 934458 | 531 | 34.1 | prophage P2W3, tail tape measure protein |
| ORF00673 | 935066 | 935701 | 636 | 34.4 | hypothetical protein |
| ORF00675 | 938377 | 937466 | 912 | 31.7 | hypothetical protein |
| ORF00676 | 939903 | 939343 | 561 | 36.5 | similar to transposase [Nostoc sp. PCC 7120] |
| ORF00677 | 940710 | 941309 | 600 | 34.7 | hypothetical protein |
| ORF00678 | 943779 | 943204 | 576 | 55.2 | Transposase (IS4 family) |
| ORF00679 | 943873 | 944775 | 903 | 38.5 | conserved hypothetical protein |
| ORF00681 | 945659 | 946297 | 639 | 32.9 | conserved hypothetical protein |
| ORF00684 | 948532 | 949287 | 756 | 33.1 | ankyrin repeat domain protein, putative |
| ORF00685 | 949310 | 950029 | 720 | 29 | hypothetical protein |
| ORF00686 | 952670 | 951885 | 786 | 38.7 | hypothetical protein |
| ORF00689 | 953574 | 954461 | 888 | 35.5 | hypothetical protein |
| ORF00692 | 958588 | 959190 | 603 | 35 | hypothetical protein |
| ORF00693 | 960120 | 959254 | 867 | 31.4 | hypothetical protein |
| ORF00696 | 963349 | 962690 | 660 | 33.2 | ankyrin repeat domain protein |
| ORF00698 | 966226 | 965411 | 816 | 37.6 | kinesin light chain |
| ORF00699 | 967271 | 967966 | 696 | 37.9 | N-acetylmuramoyl-L-alanine amidase, putative |
| ORF00702 | 973327 | 972521 | 807 | 33.6 | hypothetical protein |
| ORF00705 | 977094 | 978008 | 915 | 36.7 | putative transposase |
| ORF00707 | 979092 | 979604 | 513 | 27.5 | hypothetical protein |
| ORF00709 | 982666 | 981737 | 930 | 29 | hypothetical protein |
| ORF00713 | 986810 | 985908 | 903 | 32.9 | hypothetical protein |
| ORF00714 | 987369 | 988202 | 834 | 33 | ABC transporter transmembrane region, putative |
| ORF00715 | 988764 | 988270 | 495 | 36.2 | transposase family protein |
| ORF00728 | 1005023 | 1004139 | 885 | 32.3 | conserved hypothetical protein |
| ORF00736 | 1013720 | 1013208 | 513 | 34.3 | transposase family protein |
| ORF00738 | 1016270 | 1015533 | 738 | 32.5 | hypothetical protein |
| ORF00743 | 1021252 | 1022079 | 828 | 33.7 | ankyrin repeat domain protein, putative |
| ORF00744 | 1025166 | 1025843 | 678 | 30.8 | hypothetical protein |
| ORF00746 | 1030124 | 1029726 | 399 | 34.8 | hypothetical protein |
| ORF00749 | 1038187 | 1037726 | 462 | 35.1 | transposase family protein |
| ORF00750 | 1039900 | 1039307 | 594 | 34.2 | hypothetical protein |
| ORF00751 | 1040356 | 1040862 | 507 | 35.9 | transposase family protein |
| ORF00753 | 1041990 | 1042664 | 675 | 34.7 | hypothetical protein |
| ORF00757 | 1048403 | 1047681 | 723 | 32.9 | hypothetical protein |
| ORF00764 | 1055378 | 1054866 | 513 | 32.9 | TnpA |
| ORF00765 | 1056006 | 1055425 | 582 | 36.1 | transposase family protein |
| ORF00769 | 1058565 | 1059086 | 522 | 36 | hypothetical protein |
| ORF00774 | 1065567 | 1064653 | 915 | 29.5 | hypothetical protein |
| ORF00775 | 1066812 | 1067744 | 933 | 33.4 | DNA invertase |
| ORF00777 | 1069535 | 1068900 | 636 | 35.7 | hypothetical protein |
| ORF00779 | 1072433 | 1071441 | 993 | 34.1 | putative ATP/GTP-binding protein |
| ORF00784 | 1080395 | 1079802 | 594 | 40.2 | hypothetical protein |
| ORF00785 | 1080573 | 1081292 | 720 | 34.7 | hypothetical protein |
| ORF00786 | 1083264 | 1084097 | 834 | 31.9 | hypothetical protein |
| ORF00787 | 1084548 | 1084159 | 390 | 39.2 | conserved hypothetical protein |
| ORF00788 | 1086485 | 1085889 | 597 | 33 | hypothetical protein |

SUPPLEMENTARY TABLE 3. Unique gene in the *w*Moj Assembly

| ORF | 5’-end | 3’end | Size | % GC | Common name |
| --- | --- | --- | --- | --- | --- |
| ORF00012 | 18968 | 19675 | 708 | 32.8 | hypothetical_protein |
| ORF00018 | 26341 | 27006 | 666 | 32.6 | hypothetical_protein |
| ORF00032 | 46844 | 46104 | 741 | 52.8 | hypothetical_protein |
| ORF00033 | 46115 | 46855 | 741 | 52.9 | hypothetical_protein |
| ORF00034 | 46119 | 46859 | 741 | 52.8 | hypothetical_protein |
| ORF00049 | 68529 | 69446 | 918 | 37.5 | Phage_tail_sheath_protein |

SUPPLEMENTARY TABLE 4. Identifiers for the Trace Archive reads from the *Wolbachia* endosymbiont of *Drosophila mojavensis*. These entries can be found in the Trace Archive entry for *D. mojavensis*.

gnl|ti|495851660

gnl|ti|495851681

gnl|ti|495851690

gnl|ti|495876541

gnl|ti|495876542

gnl|ti|495876695

gnl|ti|495876696

gnl|ti|495876851

gnl|ti|495876852

gnl|ti|495876897

gnl|ti|495877020

gnl|ti|495877036

gnl|ti|495877037

gnl|ti|495877101

gnl|ti|495877199

gnl|ti|495877433

gnl|ti|495877436

gnl|ti|495877532

gnl|ti|495877695

gnl|ti|495877851

gnl|ti|495877852

gnl|ti|495878018

gnl|ti|495878048

gnl|ti|495878049

gnl|ti|495878177

gnl|ti|495878202

gnl|ti|495878203

gnl|ti|495878252

gnl|ti|495878314

gnl|ti|495878315

gnl|ti|495878384

gnl|ti|495878385

gnl|ti|495878501

gnl|ti|495878502

gnl|ti|495928296

gnl|ti|495928297

gnl|ti|495928429

gnl|ti|495928987

gnl|ti|495929030

gnl|ti|495929154

gnl|ti|495929177

gnl|ti|495929204

gnl|ti|495929528

gnl|ti|495929780

gnl|ti|495929978

gnl|ti|495930204

gnl|ti|495930205

gnl|ti|495930358

gnl|ti|495930408

gnl|ti|495930430

gnl|ti|495930586

gnl|ti|495930617

gnl|ti|495930619

gnl|ti|496020010

gnl|ti|496020080

gnl|ti|496020269

gnl|ti|496020308

gnl|ti|496020639

gnl|ti|496020640

gnl|ti|496021034

gnl|ti|496021311

gnl|ti|496021497

gnl|ti|496021783

gnl|ti|496021828

gnl|ti|496021866

gnl|ti|496021867

gnl|ti|496021872

gnl|ti|496021873

gnl|ti|496022072

gnl|ti|496062254

gnl|ti|496062344

gnl|ti|496062409

gnl|ti|496062515

gnl|ti|496062516

gnl|ti|496062846

gnl|ti|496062847

gnl|ti|496062849

gnl|ti|496062850

gnl|ti|496063037

gnl|ti|496063055

gnl|ti|496063188

gnl|ti|496063189

gnl|ti|496063220

gnl|ti|496063221

gnl|ti|496063272

gnl|ti|496063273

gnl|ti|496063294

gnl|ti|496063298

gnl|ti|496063299

gnl|ti|496063409

gnl|ti|496063726

gnl|ti|496063727

gnl|ti|496063854

gnl|ti|496063855

gnl|ti|496063878

gnl|ti|496063879

gnl|ti|496064062

gnl|ti|496064063

gnl|ti|496064098

gnl|ti|496064099

gnl|ti|496064210

gnl|ti|496064211

gnl|ti|496064392

gnl|ti|496064393

gnl|ti|496064472

gnl|ti|496064473

gnl|ti|496064532

gnl|ti|496064533

gnl|ti|496064554

gnl|ti|496064555

gnl|ti|496064694

gnl|ti|496064695

gnl|ti|496064700

gnl|ti|496064701
